# Digital solutions and the future of recovery after critical illness

**DOI:** 10.1097/MCC.0000000000001075

**Published:** 2023-08-09

**Authors:** Louise Rose, Christopher E. Cox

**Affiliations:** a Faculty of Nursing, Midwifery and Palliative Care, King's College London, London, UK; b Department of Medicine, Duke University, Durham, North Carolina, USA

**Keywords:** critical care, digital, psychological, recovery, rehabilitation

## Abstract

**Purpose of review:**

Digital technologies may address known physical and psychological barriers to recovery experienced by intensive care survivors following hospital discharge and provide solutions to care fragmentation and unmet needs. The review highlights recent examples of digital technologies designed to support recovery of survivors of critically illness.

**Recent findings:**

Despite proliferation of digital technologies supporting health in the community, there are relatively few examples for intensive care survivors. Those we identified included web-based, app-based or telemedicine-informed recovery clinics or pathways offering services, including informational resources, care planning and navigation support, medication reconciliation, and recovery goal setting. Digital interventions supporting psychological recovery included apps providing adaptive coping skills training, mindfulness, and cognitive behavioural therapy. Efficacy data are limited, although feasibility and acceptability have been established for some. Challenges include difficulties identifying participants most likely to benefit and delivery in a format easily accessible to all, with digital exclusion a resultant risk.

**Summary:**

Digital interventions supporting recovery comprise web or app-based recovery clinics or pathways and digital delivery of psychological interventions. Understanding of efficacy is relatively nascent, although several studies demonstrate feasibility and acceptability. Future research is needed but should be mindful of the risk of digital exclusion.

## INTRODUCTION

Survivors of an ICU admission experience numerous physical, psychological, and cognitive sequalae collectively known as post intensive care syndrome (PICS) that are highly prevalent and persistent [1^▪▪^]. Furthermore, fragmentation of healthcare services during recovery from critical illness is a common phenomenon [2]. This results in a mismatch between healthcare services needed and those received, information loss, treatment omissions, and hospital readmission [3–5]. In some countries such as the UK, clinical services to support recovery from critical illness are reasonably well established [6]. However, patients generally do not receive ICU recovery clinic services until 2–3 months after hospital discharge, if at all. This results in multiple unmet needs, including timely provision of assistive devices, access to services to support mental health, appropriate medication management, assistance with government and community health and social care programmes, and access to rehabilitation therapies [7^▪^,8^▪^]. Multiple unmet needs result in a challenging experience for patients and families that negatively influences recovery potential as well as time to recovery.

Digital technologies comprise electronic tools (cameras, sensors), systems and platforms (software, mobile apps), devices (tablets, smartphones), and resources (websites) that generate, store, or process data. These technologies may provide a solution to care fragmentation and unmet needs immediately following hospital discharge, enabling individualized patient and family-centred care. Digital solutions may improve patient access to appropriate care providers in a timely and cost-effective manner. Although digital technologies as a part of telemedicine and telerehabilitation services have been used extensively in the management of chronic diseases such as Chronic Obstructive Pulmonary Disease (COPD) and congestive heart failure [9], we are only just starting to see examples that facilitate rehabilitation and recovery from critical illness. In this brief review, we will outline recent examples of the use of digital technologies to support both physical and psychological recovery of ICU survivors. 

**Box 1:**
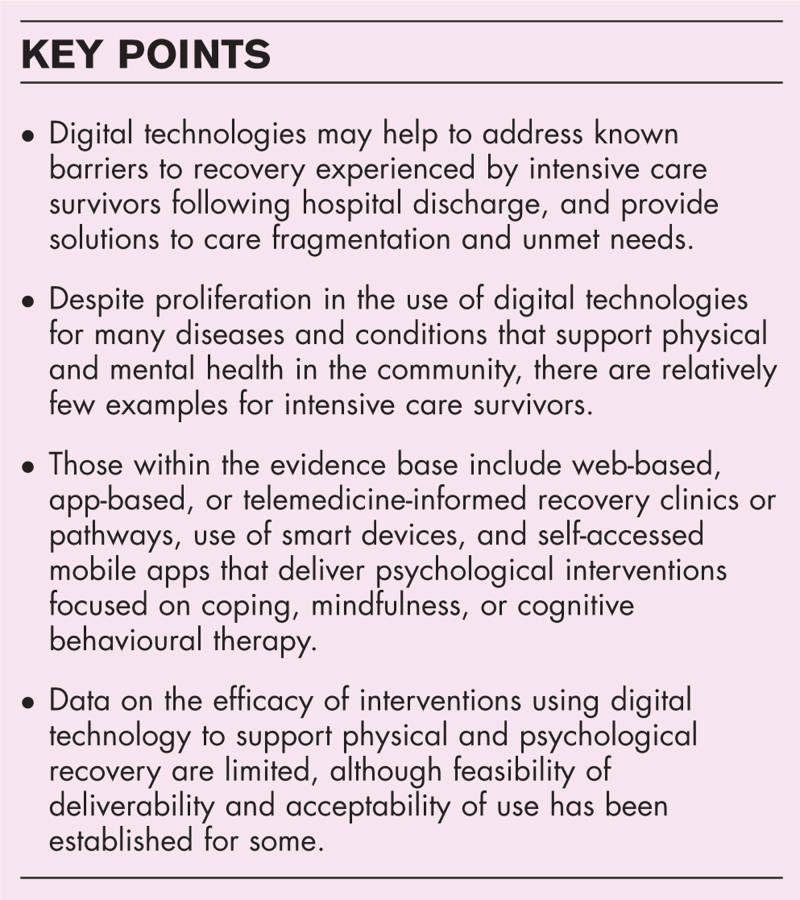
no caption available

### Digital technology-based interventions to promote physical recovery

The COVID-19 pandemic has significantly accelerated the adoption of digital technologies throughout the world and across many patient diseases and conditions. Although there are some examples of digital recovery clinics or programmes that predated the pandemic, many existing ICU recovery clinic services were forced by necessity to adapt to virtual service delivery by video conferencing due to social distancing mandates aimed at reducing virus transmission. Kovaleva *et al*. [10] described a telemedicine ICU recovery clinic in the USA commencing in 2019 that comprised video conferenced clinic visits at 3 and 12 weeks with a team, including a pharmacist, ICU physician, and a neuropsychologist. These visits focused on medication reconciliation and counselling, remote medical examination, an ICU debrief, and cognitive and mental health screening. Qualitative data indicated recipients found this approach to be acceptable, convenient, time-saving, and to facilitate thorough discussions. Another example of a web-based recovery program, ICUTogether, was developed by Ewens *et al*. [11]. This program is accessible via smart devices or computer and provides a range of informational resources, including those relating to exercise, sleep, and nutrition tailored to data provided by a patient on their current symptoms. Symptom data are also reviewed to ascertain if further interventions are needed. A randomized controlled trial (RCT) of this program is currently underway in Australia.

The USA-based STAR program for sepsis survivors [12] uses a centrally based sepsis nurse navigator who provides individualized support for care planning, self-management, follow up, and engagement until 30 days after hospital discharge. Although communication between the nurse navigator and the patient was predominantly by telephone, video conferencing and the electronic medical record were also used to enable coordination and monitoring of patients. In a RCT of 691 participants, the STAR program reduced 30-day mortality or hospital readmission compared with routine transition support and outpatient care [13^▪▪^].

**FIGURE 1:**
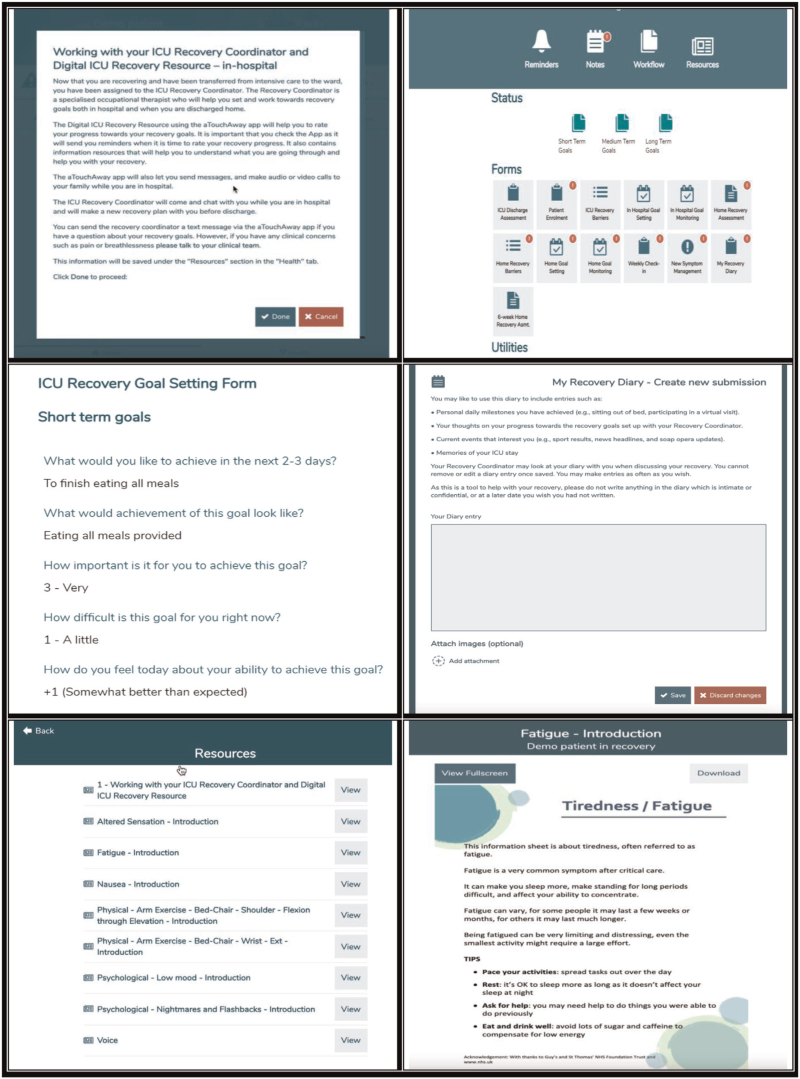
Screenshots of elements of the digital ICU Recovery Pathway. Reprinted from [15^▪^].

During the pandemic, our group Life Lines (https://www.kingshealthpartners.org/our-work/lifelines/information-for-families/information-on-the-digital-icu-recovery-pathway) designed and implemented an ICU digital recovery pathway into a large ICU recovery service in a tertiary centre in the UK. The pathway is hosted in the e-platform aTouchAway (Aetonix, Canada) and comprises e-forms for the assessment of baseline status and barriers to recovery; setting of individualized recovery goals; patient self-reported e-monitoring of goal achievement with automated reminders; provision of e-resources tailored to recovery barriers; patient recovery e-diary; tailored activity reminders; note function enabling the recovery coordinator to document patient encounters with optional electronic medical record upload; and (8) two-way digitally secure text, audio, and video communication between the patient, nominated family members, and the recovery coordinator (Fig. 1). Selection of goals is based on Goal Attainment Scaling (GAS) [14]. It includes discussion and documentation of what goal achievement would look like and the patient's perceptions of goal importance, difficulty, and ability to attain the goal. Initial evaluation of the pathway indicates the pathway is feasible to deliver and has good acceptability ratings [15^▪^].

Telemedicine and telerehabilitation programmes also have been introduced for COVID-19 survivors, many of whom have experienced an ICU admission. In an Italian study, Bernocchi *et al*. [16] reported on the adaptation of their existing telemedicine services for patients with COPD or chronic heart failure to meet the needs of COVID-19 survivors. Their 3-month programme comprises a standardized interview to assess the patient's clinical condition followed by tailored advice by telephone or video conferencing. Patients are also provided with a pulse oximeter on hospital discharge to monitor their oxygen saturation during rest and exercise. Evaluation of the programme in 130 patients demonstrated a reduction in symptoms, improvement in the physical aspect of health-related quality of life, and high programme satisfaction.

Capin *et al*. [17] developed a comprehensive multicomponent app-facilitated telerehabilitation programme for COVID-19 survivors, including those with an ICU admission. This includes provision of a tablet, Fitbit, automated blood pressure cuff, pulse oximeter, ankle weights and resistance bands with 12 individualized exercise, coaching and motivational interviewing sessions via video, and access to the Health in Motion app. An initial assessment of feasibility demonstrated excellent adherence despite the intensive nature of this intervention. Hunter *et al*. [18^▪^] have used smartwatches to measure step count and heart rate for up to 1 year in a cohort of 50 ICU survivors who had severe COVID-19 recruited from eight centres. In one centre, the smartwatch data were provided for a multiprofessional team review. Data demonstrated an increase in step count and a reduction in resting heart rate from baseline to 1 year, with good fidelity and positive perceptions in terms of the influence of the smartwatch on their recovery.

### Digital technology-based interventions to promote self-management of psychological recovery

The stresses of critical illness promote psychological distress symptoms, including depression [19], anxiety [20], and posttraumatic stress disorder (PTSD) [21] that are common and persistent. However, there are several challenges to delivering interventions to address psychological symptoms of ICU survivors. Routine screening is uncommon, access to mental healthcare is challenging and geographically inconsistent (particularly for people from racially and ethnically minoritized groups [22,23]), and therapy frequently includes medications or in-person therapist visits [24]. In contrast, approaches that patients generally prefer includes easily accessible therapy available from home, content that reflects their individual experiences, and non-pshrmacological interventions due to concerns about polypharmacy and side effects [25].

As yet, there are few examples of effective interventions that alleviate ICU survivor distress. Rigorously conducted trials demonstrate no effect on PTSD, depression, or anxiety symptoms using either ICU-based interventions (e.g., music therapy [26], nurse-led preventive psychological intervention [27], or ICU diaries [28] (now available in digital formats) or interventions following hospital discharge (e.g., aforementioned follow-up clinics/service [29]). Challenges include difficulties associated with directing an intervention towards those who are most likely to benefit, recognizing that some patients may be too ill and some perhaps too well. Another challenge is delivering an intervention in an easily accessible format with the risk of introducing digital exclusion. Such exclusion may be due to demographic, socioeconomic, or illness circumstances that prevent access to technology or lack of familiarity or comfort with its use. These challenges were encountered during a multicentre RCT examining a telephone-based adaptive coping skills intervention. On average, only half the calls were completed thereby limiting the intervention dose, with substantial dropout due to illness [30].

**FIGURE 2:**
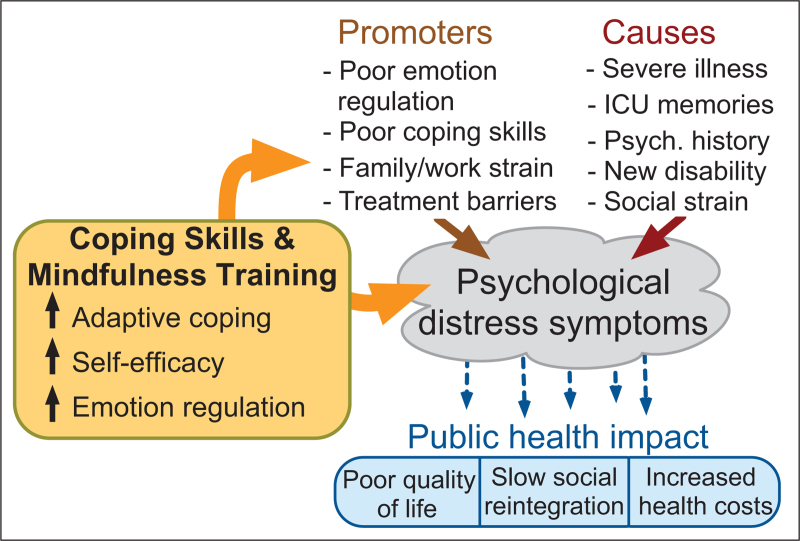
Conceptual model for action of adaptive coping skills and mindfulness training interventions.

**FIGURE 3:**
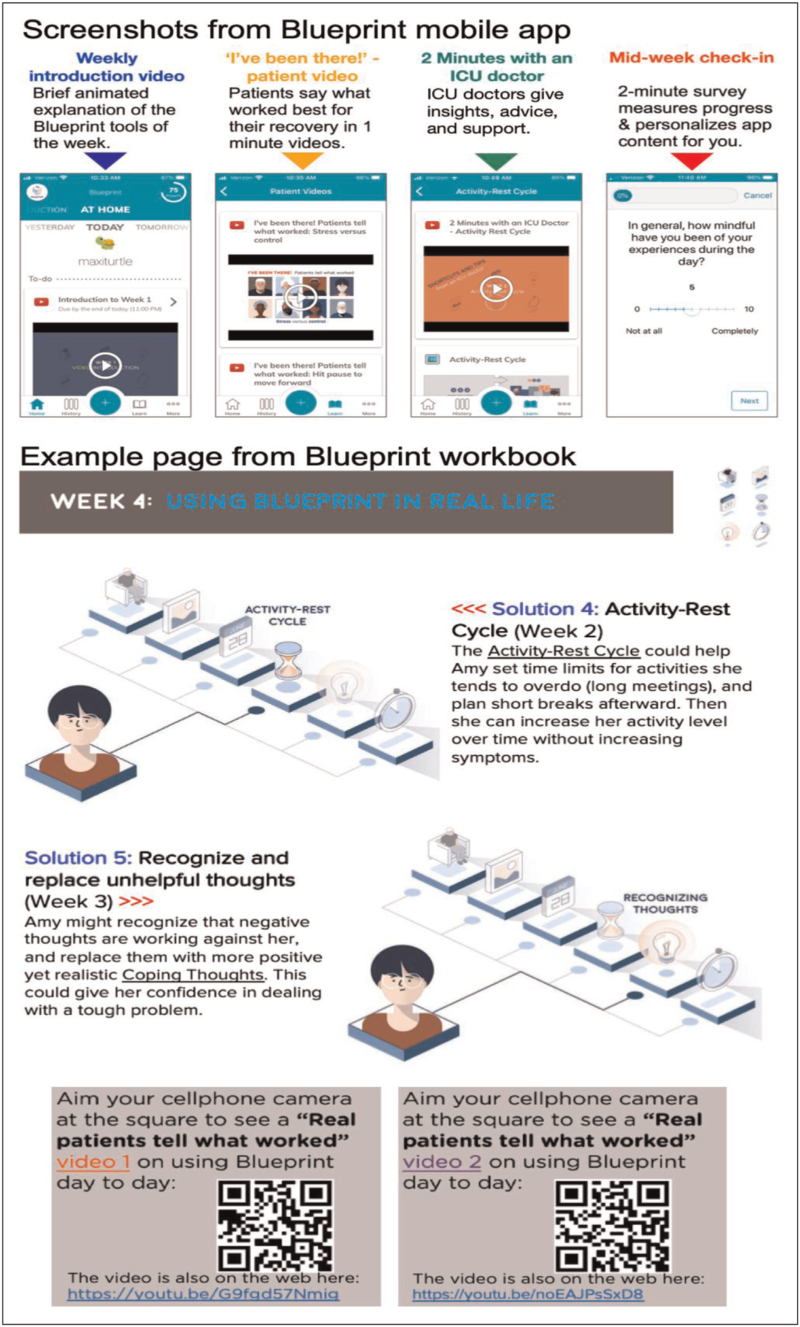
Screenshots of Blueprint adaptive coping skills training mobile app and workbook.

Although there are numerous more generalized digital interventions promoting self-management of psychological distress symptoms, there are few examples designed specifically for ICU survivors following hospital discharge (Fig. 2). One example is an adaptive coping skills training intervention called the Blueprint mobile app that evolved from the aforementioned telephone-based intervention. Blueprint is a self-directed, month-long programme with unique content arranged in four thematic weeks accessed using any digital device. Content includes short videos by ICU survivors describing coping strategies, videos in which clinicians normalize the recovery process and provide context for adaptive coping, and interactive exercises illustrating each week's coping skills (Fig. 3). A pilot RCT [31^▪^] comparing the Blueprint mobile app intervention to usual care found good adherence, with participants experiencing improvements in depression and anxiety symptoms at 3 months. An efficacy-focused trial is now required before broader adoption.

Another example of a digital intervention to address psychological distress of ICU survivors is the month-long, self-directed mobile app-based mindfulness Lift intervention. In a pilot RCT [32] with 80 participants, the Lift mobile app reduced psychological distress symptoms compared to a control group receiving a web-based critical illness education programme. Importantly, 85% of participants randomized to the Lift app received 100% of the intervention dose. Currently, there is a multicentre trial underway [33] that compares eight different versions of Lift. To determine which version is optimal, these differ in terms of varying configurations of dose, response to increasing symptoms over time, and presence or absence of a therapist kick-off call.

Other recent trials of digital technology-based interventions to promote psychological recovery include virtual reality and internet-based cognitive behavioural therapy. Vlake *et al*. [34^▪^] found no effect on psychological distress in a trial (89 participants) of a 14-min informational video exposing the patient to the ICU environment with voice-over explanations delivered using virtual reality during an ICU recovery clinic visit. Gawlytta *et al*. [35] conducted a trial targeting dyads of sepsis survivors with a presumptive PTSD diagnosis and their spouse. They found no effect of an internet-based, therapist-led partner-assisted cognitive-behavioural writing therapy comprising two 50 min internet-based writing assignments/week/5 weeks but were only able to recruit 25 dyads of the planned 98. Although these interventions have not demonstrated efficacy in decreasing psychological distress, they are likely under-powered and do however provide important data informing understanding of the acceptability of these digital interventions.

## CONCLUSION

Survivors of an ICU admission experience numerous physical, psychological, and cognitive sequalae for which digital technologies may offer alternate interventions and delivery methods to traditional in-person recovery clinics. These in-person clinics in themselves are not offered consistently across regions or countries, if at all. Digital interventions described in the evidence base primarily comprise web or app-based recovery clinics or pathways addressing a range of ICU survivor needs; digital delivery of psychological interventions such as coping, mindfulness, or cognitive behavioural therapy; and use of virtual reality. Evidence in relation to the efficacy of such interventions is relatively nascent although several studies demonstrate feasibility and acceptability. Future research is needed to establish efficacy of digital technologies in supporting ICU survivor recovery but should also be mindful of the associated risk of digital exclusion.

## Acknowledgements

*None.*

### Financial support and sponsorship

*None.*

### Conflicts of interest

*L.R. is a co-founder of Life Lines, a philanthropic COVID-19 rapid response project that received charitable donations to enable provision of over 1400 4G enabled Android tablets and a bespoke virtual visiting solution to ICUs across the UK. Major philanthropic contributors to Life Lines include Google, True Colours, and the Gatsby Trust. British Telecom contributed in-kind time and resources to facilitate the supply of 4G-enabled tablets to UK ICUs.*

*Life Lines also received funding from the Guy's and St Thomas Hospital Foundation Trust for the development and implementation of the ICU digital recovery pathway described in this paper. L.R. has no financial or commercial interests in Life Lines or the ICU digital recovery pathway.*

*C.C. received grant funding from the National Institutes of Health. He has no other financial or commercial interests.*

## REFERENCES AND RECOMMENDED READING

Papers of particular interest, published within the annual period of review, have been highlighted as:

▪ of special interest
▪▪ of outstanding interest

## References

[1▪▪] Herridge M Azoulay É. Outcomes after critical illness. N Engl J Med 2023; 388:913–924. 36884324 10.1056/NEJMra2104669

[2] Taylor S Chou S Sierra M. Association between adherence to recommended care and outcomes for adult survivors of sepsis. Ann Am Thorac Soc 2020; 17:89–97. 31644304 10.1513/AnnalsATS.201907-514OC PMC6944350

[3] Stelfox H Leigh J Dodek P. A multicenter prospective cohort study of patient transfers from the intensive care unit to the hospital ward. Intensive Care Med 2017; 43:1485–1494. 28852789 10.1007/s00134-017-4910-1

[4] Misak C Herridge M Ely E. Patient and family engagement in critical illness. Crit Care Med 2021; 49:1389–1401. 34091487 10.1097/CCM.0000000000005136

[5] Deb P Murtaugh C Bowles K. Does early follow-up improve the outcomes of sepsis survivors discharged to home healthcare? Med Care 2019; 57:633–640. 31295191 10.1097/MLR.0000000000001152 PMC11648946

[6] Connolly B Milton-Cole R Adams C. Recovery, rehabilitation and follow-up services following critical illness: an updated UK national cross-sectional survey and progress report. BMJ Open 2021; 11:e052214. 10.1136/bmjopen-2021-052214 PMC8491421 34607869

[7▪] Bose S Groat D Dinglas V. Association between unmet nonmedication needs after hospital discharge and readmission or death among acute respiratory failure survivors: a multicenter prospective cohort study. Crit Care Med 2023; 51:212–221. 36661449 10.1097/CCM.0000000000005709

[8▪] Brown S Dinglas V Akhlaghi N. Association between unmet medication needs after hospital discharge and readmission or death among acute respiratory failure survivors: the addressing postintensive care syndrome (APICS-01) multicenter prospective cohort study. Crit Care 2022; 26:6. 34991660 10.1186/s13054-021-03848-3 PMC8738999

[9] Ambrosino N Vagheggini G Mazzoleni S Vitacca M. Telemedicine in chronic obstructive pulmonary disease. Breathe 2016; 12:350–356. 28210321 10.1183/20734735.014616 PMC5297949

[10] Kovaleva M Jones A Kimpel C. Patient and caregiver experiences with a telemedicine intensive care unit recovery clinic. Heart Lung 2023; 58:47–53. 36399862 10.1016/j.hrtlng.2022.11.002 PMC9992018

[11] Ewens B Myers H Whitehead L. A web-based recovery program (ICUTogether) for intensive care survivors: protocol for a randomized controlled trial. JMIR Res Protoc 2019; 8:e10935. 30664478 10.2196/10935 PMC6354195

[12] Taylor S Rios A Kowalkowski M. Translating postsepsis care to post-COVID-19 care. The case for a virtual recovery program. Ann Am Thorac Soc 2021; 18:938–941. 33570480 10.1513/AnnalsATS.202006-649IP PMC8456723

[13▪▪] Taylor S Murphy S Rios A. Effect of a multicomponent sepsis transition and recovery program on mortality and readmissions after sepsis: the improving morbidity during postacute care transitions for sepsis randomized clinical trial. Crit Care Med 2022; 50:469–479. 34534130 10.1097/CCM.0000000000005300 PMC10229099

[14] Ashford S Turner-Stokes L. Goal attainment for spasticity management using botulinum toxin. Physiother Res Int 2006; 11:24–34. 16594313 10.1002/pri.36

[15▪] Rose L Apps C Brooks K. Novel digitally enabled care pathway to support postintensive care recovery and goal attainment following critical illness. BMJ Innov 2022; 8: 10.1136/bmjinnov-2021-000842

[16] Bernocchi P Bonometti F Serlini M. Telehealth and telecare: a real-life integrated experience in the COVID-19 pandemic. Telemed J E Health 2022; 28:720–727. 34402684 10.1089/tmj.2021.0181

[17] Capin J Jolley S Morrow M. Safety, feasibility and initial efficacy of an app-facilitated telerehabilitation (AFTER) programme for COVID-19 survivors: a pilot randomised study. BMJ Open 2022; 12:e061285. 10.1136/bmjopen-2022-061285 PMC9329728 35882451

[18▪] Hunter A Leckie T Coe O. Using smartwatches to observe changes in activity during recovery from critical illness following COVID-19 critical care admission: 1-year, multicenter observational study. JMIR Rehabil Assist Technol 2022; 9:e25494. 35417402 10.2196/25494 PMC9063865

[19] Rabiee A Nikayin S Hashem M. Depressive symptoms after critical illness: a systematic review and meta-analysis. Crit Care Med 2016; 44:1744–1753. 27153046 10.1097/CCM.0000000000001811 PMC7418220

[20] Nikayin S Rabiee A Hashem M. Anxiety symptoms in survivors of critical illness: a systematic review and meta-analysis. Gen Hosp Psychiatry 2016; 43:23–29. 27796253 10.1016/j.genhosppsych.2016.08.005 PMC5289740

[21] Parker A Sricharoenchai T Raparla S. Posttraumatic stress disorder in critical illness survivors: a metaanalysis. Crit Care Med 2015; 43:1121–1129. 25654178 10.1097/CCM.0000000000000882

[22] Olfson M Zuvekas S McClellan C. Racial-ethnic disparities in outpatient mental healthcare in the United States. Psychiatr Serv 2023; 74:674–683. 36597696 10.1176/appi.ps.20220365

[23] Kuehn B. Clinician shortage exacerbates pandemic-fueled ‘mental health crisis’. JAMA 2022; 327:2179–2181. 35612873 10.1001/jama.2022.8661

[24] Tully P Ang S Lee E. Psychological and pharmacological interventions for depression in patients with coronary artery disease. Cochrane Database Syst Rev 2021; 12:CD008012. 34910821 10.1002/14651858.CD008012.pub4 PMC8673695

[25] Dwight-Johnson M Sherbourne C Liao D Wells K. Treatment preferences among depressed primary care patients. J Gen Intern Med 2000; 15:527–534. 10940143 10.1046/j.1525-1497.2000.08035.x PMC1495573

[26] Chlan L Weinert C Heiderscheit A. Effects of patient-directed music intervention on anxiety and sedative exposure in critically ill patients receiving mechanical ventilatory support: a randomized clinical trial. JAMA 2013; 309:2335–2344. 23689789 10.1001/jama.2013.5670 PMC3683448

[27] Wade D Mouncey P Richards-Belle A. Effect of a nurse-led preventive psychological intervention on symptoms of posttraumatic stress disorder among critically Ill patients: a randomized clinical trial. JAMA 2019; 321:665–675. 30776295 10.1001/jama.2019.0073 PMC6439605

[28] Brandeo Barreto B Luz M Rios M. The impact of intensive care unit diaries on patients’ and relatives’ outcomes: a systematic review and meta-analysis. Crit Care 2019; 23:411. 31842929 10.1186/s13054-019-2678-0 PMC6916011

[29] Schofield-Robinson O Lewis S Smith A McPeake J. Follow-up services for improving long-term outcomes in intensive care unit (ICU) survivors. Cochrane Database Syst Rev 11:CD012701. 10.1002/14651858.CD012701.pub2 PMC6517170 30388297

[30] Cox C White D Hough C. Effects of a personalized web-based decision aid for surrogate decision makers of patients with prolonged mechanical ventilation: a randomized clinical trial. Ann Intern Med 2019; 170:285–297. 30690645 10.7326/M18-2335 PMC7363113

[31▪] Cox C Kelleher S Parish A. Feasibility of mobile app-based coping skills training for cardiorespiratory failure survivors: the blueprint pilot randomized controlled trial. Ann Am Thorac Soc 2023; 20:10.1513/AnnalsATS.202210-890OC PMC10257028 36603136

[32] Cox C Hough C Jones D. Effects of mindfulness training programmes delivered by a self-directed mobile app and by telephone compared with an education programme for survivors of critical illness: a pilot randomised clinical trial. Thorax 2019; 74:33–42. 29793970 10.1136/thoraxjnl-2017-211264 PMC6460929

[33] Cox C Olsen M Gallis J. Optimizing a self-directed mobile mindfulness intervention for improving cardiorespiratory failure survivors’ psychological distress (LIFT2): design and rationale of a randomized factorial experimental clinical trial. Contemp Clin Trials 2020; 96:106119. 32805434 10.1016/j.cct.2020.106119 PMC7428440

[34▪] Vlake J van Bommel J Wils E. Intensive care unit-specific virtual reality for critically ill patients with COVID-19: multicenter randomized controlled trial. J Med Internet Res 2022; 24:e32368. 34978530 10.2196/32368 PMC8812141

[35] Gawlytta R Kesselmeier M Scherag A. Internet-based cognitive-behavioural writing therapy for reducing posttraumatic stress after severe sepsis in patients and their spouses (REPAIR): results of a randomised-controlled trial. BMJ Open 2022; 12:e050305. 10.1136/bmjopen-2021-050305 PMC8915321 35264337

